# Evolution, disciplinary integration, and frontiers of multi-omics and single-cell technologies in periodontology: a bibliometric analysis (2000–2025) with an early-2026 frontier update

**DOI:** 10.3389/fcimb.2026.1875551

**Published:** 2026-09-14

**Authors:** Rong Li, Fei Du, Junli He, Zhijuan Luo, Rongyu Yang, Jianguo Liu, Yanchun Wang, Cuiling Zhou, Min Huang, Liming He

**Affiliations:** 1 Department of Stomatology, The First Affiliated Hospital of Dali University, Dali, Yunnan, China; 2 College of Clinical Medicine, Dali University, Dali, Yunnan, China; 3 Department of Stomatology, Yan’an Hospital of Kunming City, Yan’an Hospital Affiliated to Kunming Medical University, Kunming, Yunnan, China

**Keywords:** bibliometrics, host-microbiome interaction, multi-omics, periodontitis, precision dentistry, single-cell RNA sequencing (scRNA-seq), systems biology

## Abstract

The field of periodontology is undergoing a paradigm shift from traditional microbiology to systems biology, driven by advancements in multi-omics and single-cell technologies. Despite the rapid surge in publications, a Web of Science Core Collection (WoSCC)-based bibliometric and scientometric analysis mapping the evolutionary trajectory and emerging frontiers of these technologies in periodontal research remains needed. We performed a bibliometric and scientometric analysis of literature published from 2000 to 2025, with records from early 2026 used only as an exploratory frontier update, using the Web of Science Core Collection (WoSCC). Data visualization and network analysis were conducted using VOSviewer, CiteSpace, and the R-bibliometrix package. Additionally, Latent Dirichlet Allocation (LDA) topic modeling was employed to extract underlying research themes from unstructured text data. A total of 559 eligible publications were identified, showing a marked growth trend. China and the United States emerged as the dominant contributors. The evolutionary trajectory was delineated into three distinct phases: foundational exploration, multi-omics integration, and the single-cell resolution era. LDA modeling identified nine core topics, focusing on local host-microbiome interactions, systemic interconnections, and periodontal regeneration-related research. Keyword bursts highlighted “single-cell RNA sequencing (scRNA-seq),” “microenvironment,” and “macrophage polarization” as active current frontiers. The field appears to be transitioning within the WoSCC-indexed literature from descriptive microbiome studies toward functional multi-omics and single-cell-resolution analysis, providing an exploratory knowledge-map-based reference for future precision diagnostics and regenerative research in periodontology.

## 1 Introduction

Periodontitis is a chronic inflammatory disease initiated by plaque biofilms, characterized by the progressive destruction of periodontal supporting tissues and is the leading cause of tooth loss in adults (Papapanou et al., 2018). The classification and diagnosis of these conditions have been standardized to provide a unified clinical framework (Armitage, 1999). This disease exhibits high heterogeneity and complexity, influenced by the colonization of specific microbial communities (Socransky et al., 1998), host immune-inflammatory responses (Offenbacher et al., 2007), genetic susceptibility, and environmental factors (Hajishengallis, 2015). The polymicrobial disruption of host homeostasis remains the central theme in understanding its pathogenesis (Darveau, 2010).

Severe periodontitis affects nearly 10% of the global population, imposing a massive oral health burden and sharing bidirectional associations with systemic diseases like diabetes and rheumatoid arthritis (Kassebaum et al., 2014). Specifically, recent evidence has linked periodontal pathogens like *Aggregatibacter actinomycetemcomitans* to the development of autoimmunity in rheumatoid arthritis (Konig et al., 2016), highlighting the profound local-systemic interface driven by chronic inflammation (Hajishengallis and Li, 2021). Traditional diagnostic methods rely primarily on clinical indices and radiography, which often fail to capture the dynamic molecular heterogeneity of the disease (Tonetti et al., 2018). Therefore, resolving the molecular networks and cellular landscapes underlying periodontitis is a core challenge.

With the development of high-throughput sequencing, multi-omics integration (genomics, transcriptomics, proteomics, and metabolomics) has provided powerful tools for decoding these mechanisms. In periodontology, these technologies are driving a paradigm shift toward systems biology. Transcriptomic studies have revealed expression profiles involved in innate immunity and tissue repair (Kebschull et al., 2014). Early large-scale transcriptomic analyses of healthy and diseased gingival tissues provided the initial blueprint for understanding these molecular shifts (Demmer et al., 2008). Proteomic analyses have identified key proteins in gingival crevicular fluid (GCF) associated with matrix degradation (Bostanci et al., 2010), while metabolomics has delineated small-molecule profiles during host-microbiome interactions (Barnes et al., 2009). Longitudinal studies have further confirmed that specific crevicular fluid biomarkers can serve as critical predictors of future disease progression (Kinney et al., 2014). These multi-dimensional data jointly construct a three-dimensional network of periodontal pathology.

However, bulk sequencing technologies acquire average signals, failing to resolve specific contributions of different cell subsets. Recent breakthroughs in single-cell RNA sequencing (scRNA-seq) have brought revolutionary opportunities for mapping cellular landscapes at single-cell resolution. Landmark studies have utilized scRNA-seq to reconstruct the human oral mucosa cell atlas and delineate distinctive immune cell profiles in periodontal health and disease (Williams et al., 2021). These high-resolution maps have revealed the critical roles of specific cell populations, such as dysregulated macrophages, in maintaining or disrupting periodontal homeostasis (Almubarak et al., 2020). Combined with spatial transcriptomics and single-cell epigenomics, it is possible to achieve cross-omics integration to define cell identity and functional states. Furthermore, pathogens like *Porphyromonas gingivalis* have been shown to directly modulate host cell cycle progression, adding an additional layer of complexity to the cellular microenvironment (Kuboniwa et al., 2008). The integration of these layers is also essential for understanding how oral dysbiosis alters the systemic metabolome (Kato et al., 2018).

Despite the rapid surge in publications, this unprecedented influx of high-dimensional data has inadvertently led to “data overload” and “knowledge fragmentation.” Traditional narrative reviews struggle to synthesize these fragmented insights into a cohesive, systems-level understanding. Therefore, advanced knowledge-mapping tools, coupled with Latent Dirichlet Allocation (LDA) topic modeling, can help extract underlying intellectual structures and research themes (Blei et al., 2003; Garfield, 2004). Computational techniques that optimize semantic coherence within these models are also useful for identifying meaningful research themes in an expanding literature base (Mimno et al., 2011; Röder et al., 2015). Importantly, the present study does not aim to conduct a conventional systematic review of clinical evidence or to evaluate treatment effectiveness. Instead, it is designed as a WoSCC-based bibliometric and scientometric analysis to map publication trends, collaboration networks, disciplinary evolution, keyword bursts, topic structures, and representative intellectual turning points. To bridge this gap, this WoSCC-based bibliometric and scientometric analysis maps the intellectual landscape of functional omics and single-cell-resolution research in periodontology from 2000 to 2025, with early-2026 records used only to explore emerging frontier signals. Accordingly, the findings of this study should be interpreted as bibliometric observations and literature-trend signals, rather than as direct evidence of biological mechanisms, diagnostic accuracy, therapeutic efficacy, or clinical utility. Specifically, we seek to address: 1) how foundational knowledge has evolved from pathogen-centric microbiology to functional multi-omics; 2) which representative intellectual turning points can be identified within the WoSCC-indexed literature; and 3) which emerging topics are associated with specific cellular subpopulations and molecular pathways.

## 2 Materials and methods

### 2.1 Data retrieval

This bibliometric and scientometric analysis was reported with reference to PRISMA to transparently document literature identification, screening, and inclusion, rather than to present the study as a conventional systematic review with clinical evidence synthesis or risk-of-bias assessment. PRISMA was used because its checklist and four-stage flow diagram improve reporting transparency and reproducibility by requiring explicit documentation of the research question, search strategy, inclusion/exclusion criteria, screening workflow, and reasons for exclusion. The literature retrieval, screening, and bibliometric-analysis workflow is presented in **Figure 1**. The complete search strategy, field tags, inclusion/exclusion criteria, and sensitivity-search terms are provided in **Supplementary Table 1**. Scientometric studies commonly rely on biomedical databases such as Web of Science (WoS), Scopus, PubMed/MEDLINE, and Embase. Although integrating several databases can increase coverage, it may also introduce duplicate records, inconsistent source metadata, and non-equivalent citation fields, which can affect co-citation, bibliographic coupling, and collaboration-network analyses. Therefore, this study selected the Web of Science Core Collection (WoSCC) as the primary data source. WoSCC has strict journal-selection standards and provides relatively complete and standardized cited-reference data, which are essential for CiteSpace, VOSviewer, and bibliometrix analyses. PubMed/MEDLINE is highly valuable for biomedical retrieval but does not provide the same complete citation-linkage structure required for deep bibliometric mapping, whereas Scopus and Embase may increase coverage but can introduce heterogeneous citation formats and duplication. Accordingly, the present work should be interpreted as a WoSCC-based bibliometric mapping rather than an exhaustive multi-database systematic review. Because the analysis was based exclusively on WoSCC, the observed collaboration networks, citation structures, publication trends, country/institution distributions, and journal patterns may partly reflect the coverage characteristics of this database. Therefore, the results represent WoSCC-indexed periodontal research rather than the entirety of global periodontal research. This limitation is further acknowledged in the Limitations section.

**Figure 1:**
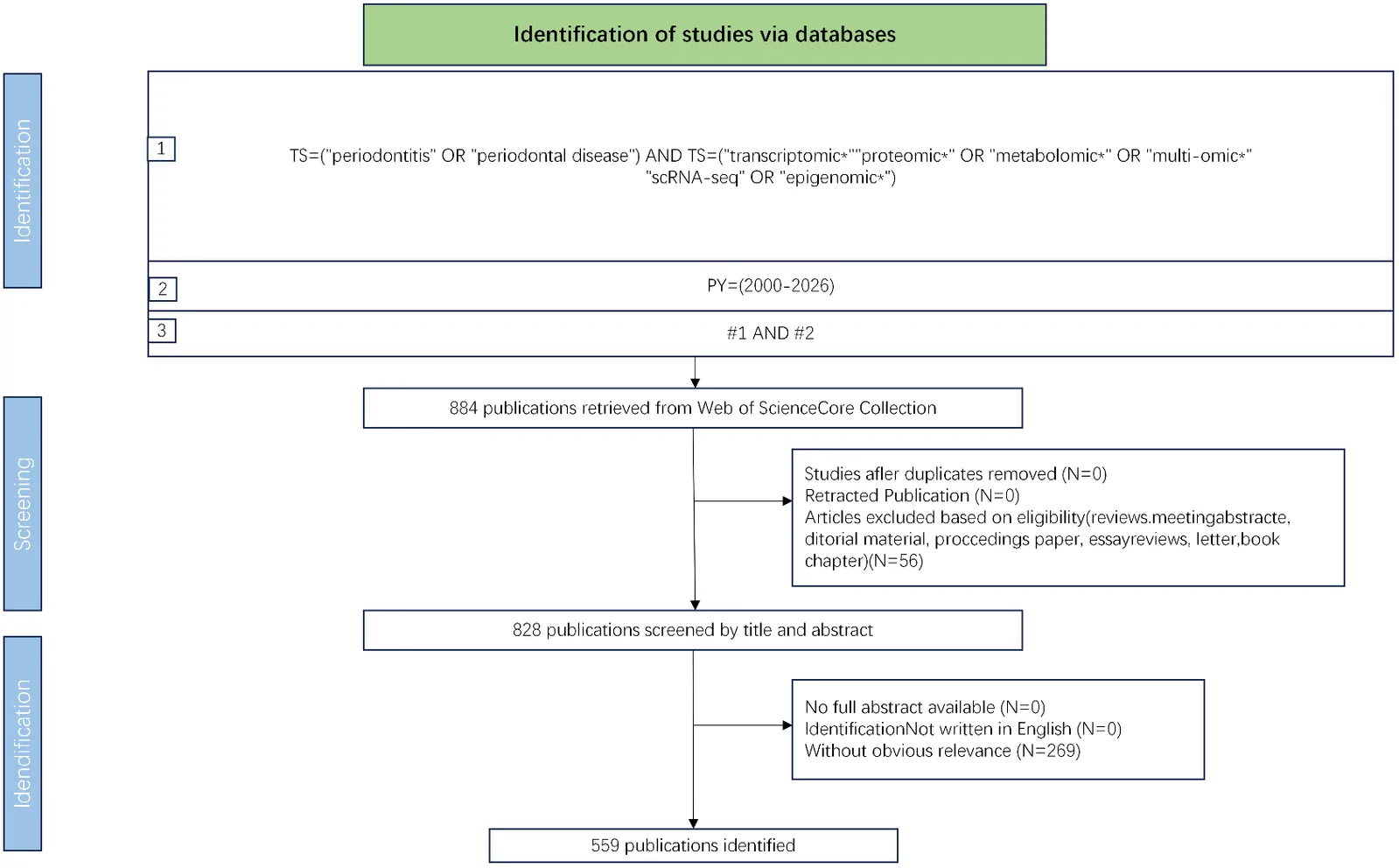
Flowchart of the systematic literature search strategy and analysis process regarding multi-omics evolution and single-cell technologies in periodontology. This image illustrates the complete workflow from database retrieval to the final analysis, including the steps for search term combinations and screening criteria.

On February 14, 2026, we conducted a search in the WoSCC database using the following keyword combinations. To improve transparency and allow readers to directly understand the retrieval strategy without consulting the **Supplementary Materials**, the exact primary search string is provided here: Search strategy (TS = Topic Search): TS = (“periodontitis” OR “periodontal disease”) AND TS = (“transcriptomic*” OR “proteomic*” OR “metabolomic*” OR “multi-omic*” OR “scRNA-seq” OR “epigenomic*”). In this search syntax, the truncation symbol “*” was used to capture variant word endings, Boolean “OR” was used to combine synonymous or related terms, Boolean “AND” was used to restrict records to those simultaneously related to periodontal disease and omics/single-cell technologies, and quotation marks were used for exact phrase searching. The complete search strategy, field tags, inclusion/exclusion criteria, and sensitivity-search terms are provided in **Supplementary Table 1**. Therefore, the search string reported in the main text represents the primary retrieval strategy, whereas **Supplementary Table 1** provides the complete technical details for reproducibility.

The primary search strategy was intentionally focused on functional host-oriented omics and single-cell-resolution technologies. Broad terms such as “genomic*” may retrieve a large number of genome-wide association studies, static genetic susceptibility studies, or structural genome-mapping studies, which are biologically important but not always aligned with the present focus on dynamic molecular phenotypes. Similarly, terms such as “metagenomic*” and “microbiome” may retrieve a large volume of descriptive microbial-community studies. Because host-microbiome interaction is central to periodontology, these terms were not dismissed as irrelevant; instead, their influence was evaluated through sensitivity checks to determine whether they substantially altered the thematic structure of the retrieved dataset. Spatial transcriptomics and broader “single-cell sequencing” terms were also considered in sensitivity analyses. Spatial transcriptomics is an important emerging frontier but remains relatively limited in large-scale periodontal multi-omics studies, while the broader term “single-cell sequencing” may retrieve single-cell DNA sequencing, single-cell ATAC-seq, or other technologies beyond the main scRNA-seq focus. Thus, the primary search retained a focused structure, while additional terms such as “genomic*,” “metagenomic*,” “microbiome,” “microbiota,” “16S rRNA,” “spatial transcriptomic*,” “single-cell sequencing,” “single cell sequencing,” and “scRNAseq” were evaluated as sensitivity-search terms rather than being treated as the primary dataset-defining search terms.

The language was restricted to English, and document types were limited to Article and Early Access, with Review and Meeting Abstract excluded to focus on original research records suitable for citation-network mapping. All search results were exported on the same day to avoid bias caused by database updates. Because data retrieval was conducted on February 14, 2026, the 2026 records represented only an early partial-year dataset. Therefore, 2025 was treated as the final complete year for longitudinal growth and development-trend analyses, whereas early-2026 records were retained only as an exploratory update for thematic and frontier analyses. The exported file formats included plain text, BibTeX, and tab-delimited files. After screening, 559 records were identified for the subsequent scientometric analysis. The detailed retrieval, screening, and bibliometric-analysis workflow is illustrated in **Figure 1**. The complete search strategy, field tags, inclusion/exclusion criteria, and sensitivity-search terms are provided in **Supplementary Table 1**.

### 2.2 Data analysis and visualization

In this study, the retrieved data were imported into CiteSpace (version 6.4.R1), VOSviewer (version 1.6.20), the bibliometrix package (version 4.3.0) in R (version 4.4.0), KH Coder software (version 3b07d), and the Online Bibliometric Analysis platform to analyze published research and generate visualization maps. VOSviewer can perform knowledge graph visualizations, such as co-authorship analysis, co-occurrence analysis, and co-citation analysis, on large-scale literature data to represent authors, journals, and other relevant information. It has been widely applied in bibliometric analysis research. Different nodes represent authors, countries, institutions, journals, and keywords. The size of the nodes indicates the corresponding citation or cited frequencies. The links between nodes represent collaboration and co-occurrence relationships. The colors of the nodes and lines represent different clusters, corresponding years, or average publication years. We also utilized CiteSpace (6.4.R1) to conduct a dual-map overlay and journal co-citation cluster analysis to determine the developmental dynamics and future trends of the research field. Additionally, an international collaboration network among countries was established using the Online Bibliometric Analysis platform.

The bibliometrix package in R was used to generate a historiographic network of references, displaying citation relationships between documents and revealing how knowledge is transmitted within the research field. Furthermore, we employed the Theme-Specific Ranking (TSR) metric to assist the exploratory selection of representative documents for historiographic mapping. TSR was developed as an author-defined, study-specific ranking approach for the present bibliometric analysis. It should not be interpreted as an externally validated, standardized, or universally applicable bibliometric indicator. TSR is a composite quantitative index used to rank the significance of literature within a particular thematic sub-domain. It was used only as a supplementary exploratory tool to support the identification of potentially representative documents, rather than to establish definitive rankings of scientific importance. The algorithmic logic of TSR integrates citation impact with semantic centrality, formulated as follows:


$$TSR_{i}=\omega_{1}\cdot Norm\;\left(LCS_{i}\right)\;+\omega_{2}\cdot Norm\;\left(TRW_{i}\right)$$

where *TSR_i_* is the final score for document i; *LCS_i_* (Local Citation Score) denotes the number of citations received by the document from within the specific dataset; and *TRW_i_* (Thematic Relevance Weight) represents semantic importance, calculated by summing the co-occurrence intensities of all core keywords associated with document i within its research cluster. The function Norm(χ) denotes min-max normalization applied to each metric to ensure scale consistency ([0, 1]). To account for the power-law distribution and positive skewness of citation data, a log-transformation [ln(χ+1)] was applied to raw LCS values before normalization. TSR was used as a study-specific composite ranking score to support the selection of representative documents for historiographic visualization; it was not intended to replace established bibliometric indicators such as total citations, local citation score, co-citation strength, or H-index. The weighting coefficients ω1 and ω2 were assigned values of 0.6 and 0.4, respectively, because historiographic mapping primarily aims to trace knowledge transmission within the dataset, while thematic relevance acts as a secondary filter to ensure that the selected papers are located at the semantic core of each theme. To reduce subjectivity, alternative weighting schemes (0.5/0.5 and 0.7/0.3) were examined in a supplementary sensitivity check, and the core set of pivotal documents remained largely stable. Nevertheless, because TSR has not been externally validated, all TSR-based findings were interpreted cautiously as supportive and exploratory indicators of potential intellectual turning points, rather than as definitive bibliometric evidence of document importance.

Topic modeling is a natural language processing (NLP) technique used to discover latent topics within public literature. Latent Dirichlet Allocation (LDA) is a widely used topic modeling method capable of processing unstructured text (Blei et al., 2003). To improve reproducibility, the LDA workflow was clarified as follows. Titles and abstracts of the 559 eligible records were extracted to construct the corpus. Text preprocessing included lowercasing, tokenization, removal of punctuation, numerals, generic stop words, and domain-general words with limited discriminatory value, as well as normalization of spelling variants and treatment of multi-word technical expressions where appropriate. Domain-specific stop words were defined to prevent overly generic terms such as “periodontitis,” “study,” or “disease” from dominating topic allocation. The document-term matrix was then generated for topic modeling using KH Coder, WordCloud, and associated R-based procedures. Because the corpus size was moderate, candidate topic numbers were mainly examined within the range of 5–15. The final number of topics was determined using multiple criteria, including model coherence, pairwise cosine distance, Kullback-Leibler divergence, Arun-2010, Cao-Juan-2009, and interpretability of representative terms and documents (Mimno et al., 2011; Röder et al., 2015). K = 9 was selected because the coherence index reached a local optimum, while the Arun-2010 and Cao-Juan-2009 metrics showed favorable or stabilizing values and the decrease in perplexity became less pronounced, indicating a practical “diminishing returns” point. This choice also avoided merging distinct themes into overly broad categories or fragmenting the corpus into excessively narrow topics. Additional topic-modeling details are provided in **Supplementary Tables S5**, **S6**. In the final stage, each topic was manually named based on the top 10 representative articles and the top 20 topic terms; this manual labeling process is acknowledged as a source of subjectivity in the Limitations section.

## 3 Results

### 3.1 Analysis of longitudinal development trends and the collaboration landscape of multi-omics evolution and single-cell technologies in periodontology

The field of multi-omics evolution and single-cell technologies in periodontology experienced three development stages (**Figure 2a**). Between 2000 and 2013, research was in a stable and gradual development period, with 77 publications and 924 total citations. During this phase, the USA ranked first in publication volume (22 papers), laying an early foundation for research in this field. Other countries, such as the UK (6 papers) and Brazil (6 papers), also participated. Between 2014 and 2020, the field entered a rapid growth phase; both publication volume (180 papers) and total citations (4,314 citations) increased substantially. The USA led in publication volume (60 papers), with China ranking second (20 papers). From 2021 to 2025, the last complete year in the dataset, the field entered a phase of sustained growth, with publication volume and total citations increasing overall. Early-2026 records were analyzed only as partial-year exploratory data and were not used to define annual growth trends. China occupied a prominent position in publication volume during the recent phase (150 papers), while the USA ranked second (69 papers). Meanwhile, more countries, including Brazil (19 papers) and Germany (17 papers), became involved, indicating broader international participation.

**Figure 2:**
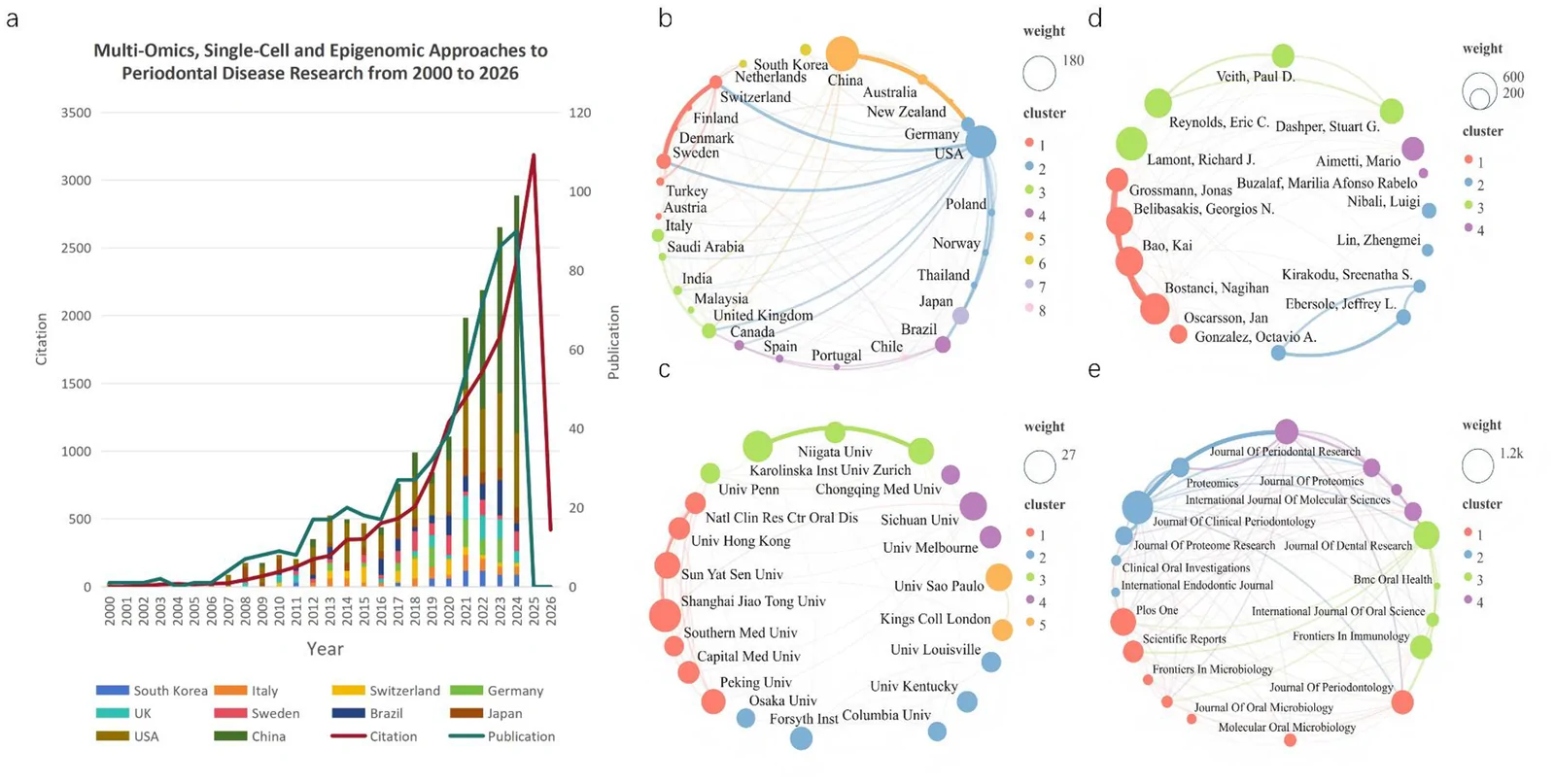
Annual publication trends and citation impact analysis in the field of multi-omics evolution and single-cell technologies in periodontology. **(a)** Shows the annual publication volume trend from 2000 to 2026 and the cumulative citation frequency of the literature, reflecting the developmental dynamics and academic impact of this research field. **(b)** National co-authorship network analysis; node size indicates the publication volume by country, and line thickness represents collaboration strength. **(c)** Institutional co-authorship network analysis; node size represents the publication volume by institution, and different colors denote distinct institutional collaboration clusters. **(d)** Author citation network analysis, illustrating the most influential scholars in the field and their academic connections. **(e)** Journal citation network analysis, displaying the core journals and the distribution of their academic impact.

Based on the national collaboration network analysis (**Figure 2b**), this research field involved 27 countries, forming 8 collaborative clusters. China was a major collaboration hub in the field of “multi-omics evolution and single-cell technologies in periodontology,” with 173 publications. Other countries, such as the USA (151 papers) and Japan (44 papers), also actively participated. In terms of collaboration strength, the links between the USA and China, as well as between Sweden and Switzerland, had a link strength of 13, making them the closest collaborative pairs in this network. Germany and the USA, and Switzerland and the USA, also engaged in collaborations, tying for second place with a link strength of 9. Additionally, collaborative associations existed between Brazil and the USA (link strength of 8), and Sweden and the USA (link strength of 8).

This research field involves 21 universities and research institutions, forming 5 collaborative clusters (**Figure 2c**). In the institutional co-authorship network, Shanghai Jiao Tong University is the most active institution in this field, with a publication volume of 26 papers. Karolinska Institutet ranks second with 23 papers, and Sichuan University ranks third with 19 papers. Institutions such as the University of Sao Paulo (18 papers) and the University of Zurich (17 papers) also contributed to the research. Regarding collaboration strength, Karolinska Institutet and the University of Zurich are the most closely connected, with a link strength of 11. The National Clinical Research Center for Oral Diseases and Shanghai Jiao Tong University rank second with a link strength of 7; the University of Sao Paulo and King’s College London rank third with a link strength of 3. Collaborative links also exist among other universities and research institutions, jointly driving the research development of multi-omics evolution and single-cell technologies in periodontology.

The field of multi-omics evolution and single-cell technologies in periodontology involves 16 core authors, forming 4 academic cluster groups (**Figure 2d**). According to the citation and publication volume data from the author citation network, N. Bostanci is the most prominent author in this field, ranking first with 13 publications and 408 citations. Bao, Kai ranks second with 13 publications and 365 citations; Belibasakis, Georgios N. ranks third with 12 publications and 342 citations. Authors such as Reynolds, Eric C., and Grossmann, Jonas also have substantial related research outputs. In terms of collaboration analysis, N. Bostanci and K. Bao have the highest collaboration frequency, with a link strength of 39; this is closely followed by the collaboration between G. N. Belibasakis and N. Bostanci, with a link strength of 32.

Combined with the journal citation network (**Figure 2e**), the field involves 20 core journals, forming 4 academic cluster groups. Based on literature citation counts and publication volumes, the Journal of Clinical Periodontology ranks first in this field with 30 publications and 1,098 citations; the Journal of Periodontal Research ranks second with 28 publications and 628 citations; and the Journal of Dental Research ranks third with 26 publications and 780 citations. Journals such as PLoS One and the Journal of Periodontology have also published a significant amount of related research, demonstrating a notable academic impact. This research area has attracted the attention of numerous scholars, forming a research landscape characterized by multidisciplinary integration.

**Table 1** presents the relevant information of the top ten countries, institutions, authors, and journals by citation counts. **Supplementary Tables S1–S4** detail the specific parameters of each node and the connections between nodes, and **Tables 2**, **3** present the detailed information on the most highly cited documents.

**Table 1:** The total number of published articles, citation counts, and average citation counts of the country, institution, author, and journal.

Ranking	Name	NC	NP	AC	TC per year	H-index	CL
Country							
1	USA	6689	151	44.3	247.74	43	***
2	China	2888	173	16.7	106.96	30	*
3	Japan	1844	44	41.9	68.30	27	ns
4	United Kingdom	1377	33	41.7	51.00	19	ns
5	Germany	1183	29	40.8	43.81	18	ns
6	Sweden	1050	34	30.9	38.89	20	ns
7	Switzerland	1017	26	39.1	37.67	18	ns
8	Brazil	851	40	21.3	31.52	17	ns
9	Canada	654	15	43.6	24.22	13	ns
10	Italy	604	24	25.2	22.37	13	ns
Institutions							
1	Columbia Univ	933	9	103.7	34.56	9	ns
2	Forsyth Inst	874	13	67.2	32.37	11	*
3	Univ Penn	605	10	60.5	22.41	7	ns
4	Univ Zurich	593	17	34.9	21.96	13	*
5	Osaka Univ	585	9	65.0	21.67	4	ns
6	Shanghai Jiao Tong Univ	567	26	21.8	21.00	14	**
7	Karolinska Inst	566	23	24.6	20.96	15	*
8	Univ Melbourne	504	12	42.0	18.67	11	ns
9	Niigata Univ	385	11	35.0	14.26	8	ns
10	Univ Louisville	372	10	37.2	13.78	7	ns
Authors							
1	Lamont, Richard J.	472	6	78.7	17.48	6	ns
2	Bostanci, Nagihan	408	13	31.4	15.11	12	**
3	Bao, Kai	365	13	28.1	13.52	12	ns
4	Reynolds, Eric C.	359	9	39.9	13.30	9	ns
5	Belibasakis, Georgios N.	342	12	28.5	12.67	11	ns
6	Dashper, Stuart G.	271	6	45.2	10.04	6	**
7	Veith, Paul D.	242	7	34.6	8.96	7	ns
8	Aimetti, Mario	240	5	48.0	8.89	5	ns
9	Grossmann, Jonas	237	9	26.3	8.78	8	ns
10	Oscarsson, Jan	150	5	30.0	5.56	5	ns
Journals							
1	Journal Of Clinical Periodontology	1098	30	36.6	40.67	20	ns
2	Journal Of Dental Research	780	26	30.0	28.89	15	**
3	Plos One	755	20	37.8	27.96	16	ns
4	Journal Of Periodontal Research	628	28	22.4	23.26	15	ns
5	Journal Of Periodontology	578	19	30.4	21.41	13	ns
6	Frontiers In Immunology	562	19	29.6	20.81	14	**
7	Scientific Reports	481	15	32.1	17.81	12	ns
8	Proteomics	386	9	42.9	14.30	9	ns
9	Journal Of Proteome Research	360	11	32.7	13.33	9	ns
10	Frontiers In Cellular And Infection Microbiology	352	12	29.3	13.04	11	ns

NP, number of publications; NC, number of citations; AC, average citations(NC/NP); TC per Year, total citation per year; H-index, Hirsch index; CL, confidence level.

**Table 2:** Most global cited documents.

Ranking	Title	Journal	Year	Total citations	TC per year
1	*Aggregatibacter actinomycetemcomitans*–induced hypercitrullination links periodontal infection to autoimmunity in rheumatoid arthritis	SCI TRANSL MED	2016	448	40.73
2	Cultivation of a human-associated TM7 phylotype reveals a reduced genome and epibiotic parasitic lifestyle	PROC NATL ACAD SCI U S A	2015	381	31.75
3	Human oral mucosa cell atlas reveals a stromal-neutrophil axis regulating tissue immunity	CELL	2021	356	59.33
4	Gingival crevicular fluid as a source of biomarkers for periodontitis	PERIODONTOL 2000	2016	314	28.55
5	UGA is an additional glycine codon in uncultured SR1 bacteria from the human microbiota	PROC NATL ACAD SCI U S A	2013	227	16.21
6	Periodontal Disease at the Biofilm–Gingival Interface	J PERIODONT	2007	186	9.30
7	Crevicular fluid biomarkers and periodontal disease progression	J CLIN PERIODONTOL	2014	177	13.62
8	Disruption of Monocyte and Macrophage Homeostasis in Periodontitis	FRONT IMMUNOL	2020	162	23.14
9	Oral Administration of *Porphyromonas gingivalis* Alters the Gut Microbiome and Serum Metabolome	MSPHERE	2018	155	17.22
10	*P. gingivalis* accelerates gingival epithelial cell progression through the cell cycle	MICROBES INFECT	2008	150	7.89

**Table 3:** Most local cited references.

Ranking	Title	Journal	Year	Citations	First author	TC per year
1	Microbial complexes in subgingival plaque	J CLIN PERIODONTOL	1998	47	SOCRANSKY SS	1.74
2	Development of a classification system for periodontal diseases and conditions	ANN PERIODONTOL	1999	40	ARMITAGE G C	1.48
3	Human oral mucosa cell atlas reveals a stromal-neutrophil axis regulating tissue immunity	CELL	2021	37	WILLIAMS DW	6.17
4	Application of label-free absolute quantitative proteomics in human gingival crevicular fluid by LC/MS E (gingival exudatome)	J PROTEOME RES	2010	36	BOSTANCI N	2.12
5	Local and systemic mechanisms linking periodontal disease and inflammatory comorbidities	NAT REV IMMUNOL	2021	36	HAJISHENGALLIS G	6.00
6	Transcriptomes in healthy and diseased gingival tissues	J PERIODONTOL	2008	34	DEMMER RT	1.79
7	Periodontitis: from microbial immune subversion to systemic inflammation	NAT REV IMMUNOL	2015	34	HAJISHENGALLIS G	2.83
8	Staging and grading of periodontitis: Framework and proposal of a new classification and case definition	J CLIN PERIODONTOL	2018	31	TONETTI MS	3.44
9	Novel protein identification methods for biomarker discovery via a proteomic analysis of periodontally healthy and diseased gingival crevicular fluid samples	J CLIN PERIODONTOL	2012	30	BALIBAN RC	2.00
10	Periodontitis: a polymicrobial disruption of host homeostasis.	NAT REV MICROBIOL	2010	29	DARVEAU RP	1.71

### 3.2 Disciplinary analysis of research on multi-omics evolution and single-cell technologies in periodontology

The dual-map overlay of journals illustrates the interdisciplinary citation network in the field of “multi-omics evolution and single-cell technologies in periodontology” (**Figure 3a**), clearly presenting its cross-disciplinary and translational characteristics. The clinical medicine journal cluster on the left side primarily focuses on fields such as periodontology, oral and maxillofacial surgery, endodontics, prosthodontics, and clinical immunology. The research focus of these journals is associated with the potential clinical relevance of multi-omics and single-cell technologies in the etiological diagnosis, disease grading, and prognosis assessment of periodontitis, as well as individualized diagnostic and treatment decision-making based on omics analysis results. The core issues they address include the correlation strength and diagnostic efficacy between the multi-omics features of periodontal tissue samples (e.g., transcriptome expression profiles, proteome differential features, and metabolome metabolite profiles) and the occurrence and development of periodontitis. They also focus on the analytical value of single-cell RNA sequencing technology in resolving the heterogeneity of immune cells and periodontal ligament cells within the periodontal microenvironment, and its possible role in informing non-surgical and regenerative periodontal treatment decisions. Furthermore, they examine the impact of different sample types (gingival crevicular fluid, periodontal ligament tissue, saliva) and omics detection technologies on the analytical accuracy of periodontology, as well as the verification and application of multi-omics-based periodontitis risk stratification models in real-world clinical diagnostic and therapeutic scenarios. This reflects a research profile characterized by a clinical problem-oriented approach and a prioritization of precision diagnosis and treatment.

**Figure 3:**
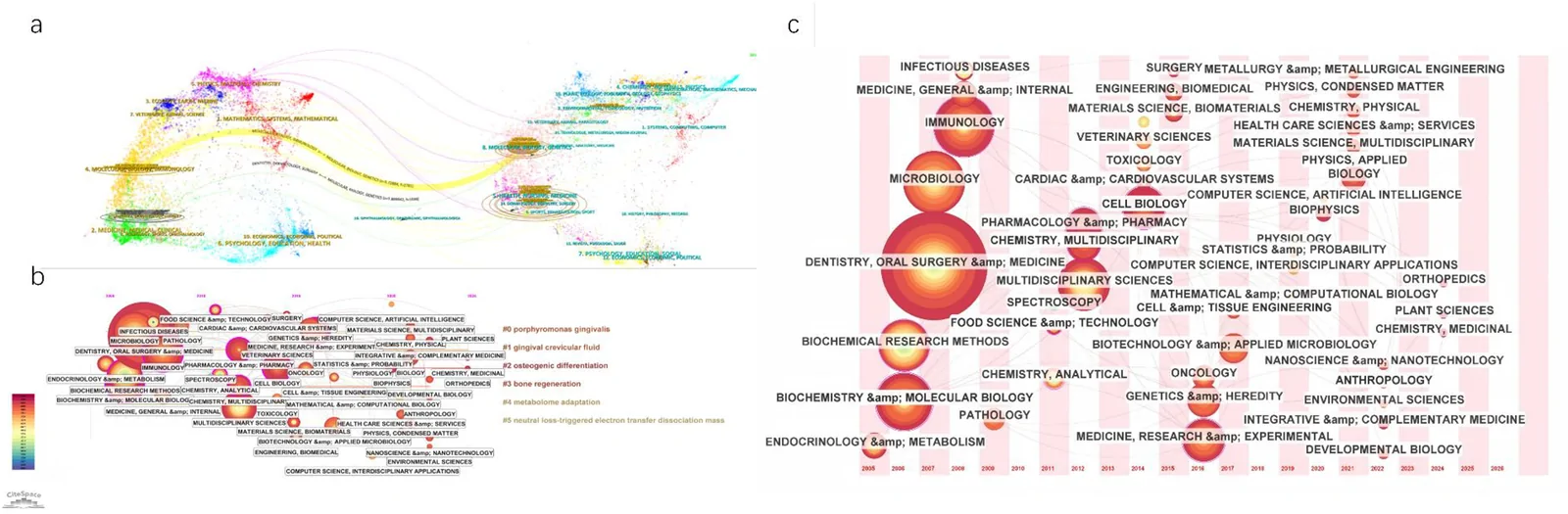
Disciplinary analysis of multi-omics evolution and single-cell technologies in periodontology. **(a)** A dual-map overlay of journals. By superimposing different disciplinary classification systems, this map visually demonstrates the interdisciplinary nature of the research field and the mutual infiltration among various disciplines. **(b)** Results of the CiteSpace disciplinary cluster analysis, highlighting the focal research directions of recent articles across different disciplines. **(c)** A time-zone map of disciplinary emergence from 2006 to 2026. Each node represents a specific discipline positioned in the year of its first appearance, with larger nodes indicating a higher frequency of occurrence.

The basic science journal cluster on the right encompasses disciplines such as bioinformatics, molecular biology, immunology, epigenetics, microbiology, and genomics. The research here emphasizes elucidating the scientific principles and technical pathways of applying multi-omics and single-cell technologies to periodontal research, from the perspectives of omics feature mining, periodontal microecological interaction mechanisms, immune regulatory pathways, and epigenetic modifications. Key focal points include the mechanistic link between periodontal tissue omics heterogeneity, periodontal pathogen colonization, and host immune responses; the principles and advantages of single-cell RNA sequencing and epigenomic technologies in capturing the dynamic changes and regulatory features of cells in the periodontal microenvironment; the corresponding relationships between omics features, periodontitis susceptibility genes, and microbiome structure (omics association analysis); and the construction of multi-omics databases, analysis model validation, and bias control methods based on multicenter cohorts. This reflects the role of basic and technical research in providing methodological and mechanistic support for clinical periodontal diagnosis and treatment.

The lines extending from the left clinical journal cluster to the right basic journal cluster represent the translational pathway originating from “the clinical diagnostic and treatment needs of multi-omics and single-cell technologies in periodontology” to driving “omics feature extraction, technology optimization, and mechanism resolution.” For example, based on the clinical observation that “ subjects with severe periodontitis are prone to relapse after treatment and have an elevated risk of systemic diseases,” researchers further carry out “studies on the mechanisms linking periodontal tissue transcriptome features with inflammatory factor expression and epigenetic modifications.” Similarly, after basic research confirms the efficacy of a certain single-cell analysis algorithm in phenotyping periodontal immune cell subsets, it is incorporated into the clinical pathway for precise periodontitis assessment. Furthermore, multi-omics association technologies are utilized to establish correlations between periodontal phenotypes, susceptibility genes, and the microbiome, providing a basis for targeted periodontal therapy and prevention. The dense intersections of the network are concentrated on key issues such as the causal correlations and diagnostic efficacy between periodontal multi-omics features and disease progression; the construction and validation of precise periodontitis phenotyping models based on multi-omics integration and single-cell technologies; the association between omics features, periodontal microecology interactions, and immune regulatory mechanisms; and the application of multi-omics-based risk stratification in individualized treatment and prevention decisions. These constitute the research core of this field.

The overall citation pattern suggests a bibliometric trend toward deeper integration, in which clinical needs may drive technological innovation and technological advances may further inform clinical research. It also reflects the mutual infiltration of traditional periodontology with modern omics technologies, single-cell sequencing, and precision medicine, illustrating the field’s trajectory toward multidisciplinary intersection, precise phenotyping, and individualized treatment. This cross-disciplinary citation network analysis helps identify emerging trends, such as the development and external validation of periodontal multi-omics joint analysis models based on multicenter, large-sample real-world data; comprehensive periodontitis assessment models integrating multi-omics with oral microbiomes and systemic disease markers; the transition from pure diagnostic phenotyping to the joint analysis of disease progression prediction, treatment response assessment, and systemic complication risk warnings; and the application of bioinformatics algorithm optimization and visualization tools in multi-omics data interpretation to enhance clinical translation efficiency and interpretability. These findings provide a visual bibliometric basis for understanding research directions, but they should not be interpreted as direct evidence that these models or technologies have already achieved validated clinical utility.

Based on the CiteSpace disciplinary cluster analysis (**Figure 3b**), the field of “multi-omics evolution and single-cell technologies in periodontology” exhibits distinct multidisciplinary intersection characteristics and a clear chronological evolutionary trajectory. The primary research clusters identified include: #0 *Porphyromonas gingivalis*, focusing on resolving the multi-omics features of the core periodontal pathogen and exploring its molecular mechanisms in periodontal microecological dysbiosis, inflammatory responses, and tissue destruction; #1 gingival crevicular fluid, utilizing technologies like metabolomics and proteomics to mine biomarkers in gingival crevicular fluid for the early diagnosis, disease monitoring, and efficacy assessment of periodontal diseases; #2 osteogenic differentiation, concentrating on the application of multi-omics technologies to elucidate the regulatory mechanisms of osteogenic differentiation in periodontal ligament stem cells and bone marrow mesenchymal stem cells, providing a theoretical basis for periodontal tissue regenerative therapy; #3 bone regeneration, integrating transcriptome, proteome, and single-cell sequencing data to reveal cellular heterogeneity and molecular regulatory networks during the repair of periodontal bone defects, driving the development of precision regenerative medicine strategies; #4 metabolome adaptation, analyzing the metabolomic features of host-microbiome interactions in the periodontal microenvironment and elucidating the role of metabolic reprogramming in periodontitis progression; and #5 neutral loss-triggered electron transfer dissociation mass, representing key technical methods in multi-omics research that provide support for the precise identification and quantification of proteins and metabolites in complex biological samples. These clusters encompass core clinical issues such as etiology, pathology, diagnostic biomarkers, and tissue regeneration, while also including methodological innovations in the application of multi-omics and single-cell technologies in periodontology, reflecting a comprehensive research layout from basic mechanism resolution to clinical translational application.

Combined with **Figure 3c**, the first phase (2008–2020) was the foundational period for multi-omics technologies and their initial clinical integration, with deep involvement from core clinical and basic technology disciplines, including Dentistry, Oral Surgery & Medicine, Immunology, Microbiology, and Biochemistry & Molecular Biology. The research direction shifted from traditional periodontal pathogen isolation, identification, and inflammatory factor detection to the exploration of molecular features at the multi-omics level. Examples include differential expression analysis of the metabolome and proteome in the gingival crevicular fluid of periodontitis subjects; research on the transcriptome response mechanisms of host cells following *Porphyromonas gingivalis* infection; early applications of basic proteomics technologies in screening periodontal disease biomarkers; and the resolution of proteomic regulatory networks in the osteogenic differentiation of periodontal ligament stem cells. Simultaneously, the integration of technical disciplines such as Biochemical Research Methods and Analytical Chemistry laid the methodological foundation for the subsequent large-scale application of multi-omics and single-cell technologies.

The second phase (2021-2025, with early-2026 records used only as an exploratory update) was characterized by intelligent technology empowerment and deeper multidisciplinary integration. Since 2021, cross-disciplinary technological integration has become an important feature, with newly added disciplines including Computer Science (Artificial Intelligence, Computational Biology), Materials Science (Biomaterials, Multidisciplinary), Pharmacology & Pharmacy, and Nanoscience & Nanotechnology. Research has expanded from molecular characterization of periodontal disease toward topics related to precise diagnosis, targeted therapy, and tissue regeneration. Examples include the construction of periodontal microenvironment cellular heterogeneity atlases based on single-cell RNA sequencing; the development of periodontitis prediction models integrating multi-omics data; the exploration of periodontal bone resorption risk stratification by radiomics and metabolomics; and the investigation of regenerative strategies based on epigenomic regulatory mechanisms. These patterns suggest a gradual transition from “single-omics characterization” to “multi-omics integrated resolution” and then toward clinically oriented translational research.

The core participating disciplines in this field include clinical core disciplines (Dentistry, Immunology, Microbiology, Oral and Maxillofacial Surgery); basic support disciplines (Biochemistry & Molecular Biology, Genetics, Developmental Biology); and technical method disciplines (Analytical Chemistry, Biochemical Research Methods, Computer Science [AI, Computational Biology], Materials Science [Biomaterials]). These disciplines intertwine, jointly supporting the complete research chain—from the resolution of periodontal microecology and host molecular mechanisms, and the screening of multi-omics biomarkers, to the application of single-cell technologies for analyzing cellular heterogeneity and developing precision diagnostic and treatment strategies. This fully demonstrates that “multi-omics evolution and single-cell technologies in periodontology” is a deeply cross-disciplinary research field centered on clinical needs and supported by advanced multi-omics and single-cell technologies.

### 3.3 Machine learning identification of nine research topics in the field of multi-omics evolution and single-cell technologies in periodontology

The machine-learning topic-modeling analysis identified nine research topics, which were grouped into four core thematic classes (**Figure 4**). Pathogen Biology and Virulence represents the core aspect of basic mechanistic resolution, focusing on elucidating the pathogenic mechanisms and virulence regulatory networks of periodontal pathogens at the multi-omics level. Within this theme, T1 Transcriptomic and Proteomic Adaptations of Periodontal Pathogens in Multi-Species Biofilms utilizes transcriptomic and proteomic technologies to analyze the gene expression and protein regulation adaptability of periodontal pathogens in multi-species biofilms, revealing their survival and pathogenic mechanisms in complex microenvironments. T4 Virulence Factors and Secretion Systems of Periodontal Pathogens: A Proteomic and Genomic Perspective integrates genomic and proteomic data to systematically identify the virulence factors and secretion systems of periodontal pathogens, elucidating the molecular mechanisms mediating host immune evasion and tissue destruction. Furthermore, research within this topic has provided foundational evidence on how specific virulence factors from keystone pathogens directly modulate host cellular processes, such as the acceleration of the gingival epithelial cell cycle (Kuboniwa et al., 2008).

**Figure 4:**
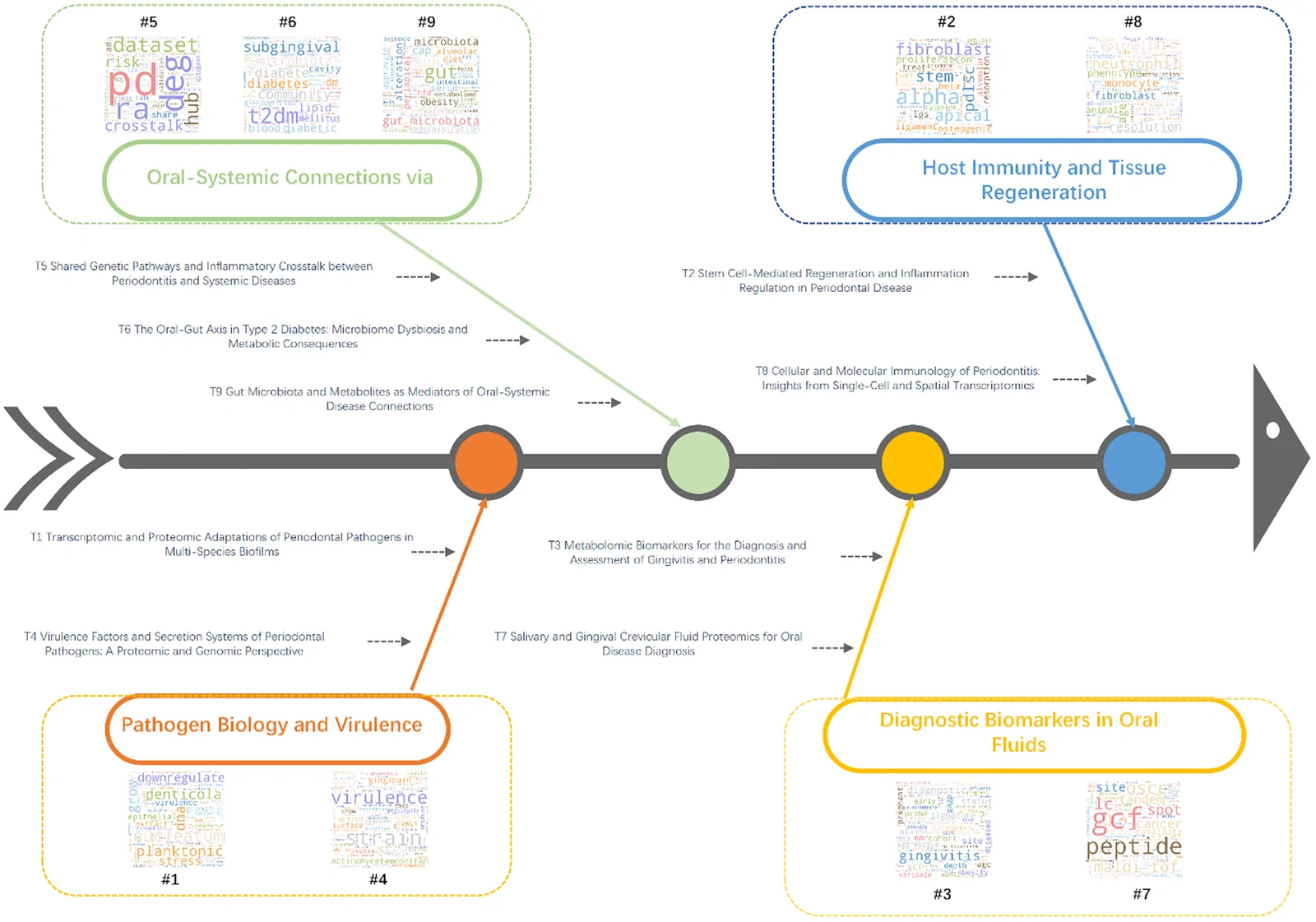
Topic modeling results for multi-omics evolution and single-cell technologies in periodontology. The results are categorized into four core classes comprising a total of nine topics, accompanied by word clouds displaying the high-weight terms for each topic.

Diagnostic Biomarkers in Oral Fluids serves as a bridge from molecular phenotypes to clinical applications, focusing on the discovery of precise diagnostic biomarkers in oral fluids using multi-omics technologies. T3 Metabolomic Biomarkers for the Diagnosis and Assessment of Gingivitis and Periodontitis employs metabolomic analysis to screen and validate metabolite biomarkers associated with gingivitis and periodontitis in oral fluids, such as gingival crevicular fluid and saliva, enabling early disease diagnosis and condition assessment. Seminal longitudinal research within this theme has critically validated the efficacy of specific crevicular fluid biomarkers in predicting and monitoring periodontal disease progression (Kinney et al., 2014). T7 Salivary and Gingival Crevicular Fluid Proteomics for Oral Disease Diagnosis utilizes proteomic technologies to systematically analyze the protein expression profiles in saliva and gingival crevicular fluid, developing protein biomarkers applicable for the non-invasive diagnosis, therapeutic monitoring, and prognostic evaluation of periodontal disease.

Oral-Systemic Connections via Multi-Omics is a crucial direction for cross-disciplinary expansion, focusing on elucidating the molecular associations and comorbidity mechanisms between periodontal disease and systemic diseases using multi-omics technologies. T5 Shared Genetic Pathways and Inflammatory Crosstalk between Periodontitis and Systemic Diseases uses genomic and transcriptomic analyses to identify shared genetic pathways and inflammatory crosstalk mechanisms between periodontal disease and systemic diseases like diabetes and cardiovascular diseases, revealing their comorbidity foundation. T6 The Oral-Gut Axis in Type 2 Diabetes: Microbiome Dysbiosis and Metabolic Consequences integrates the metagenomic data of oral and gut microbiota with host metabolomic data to analyze the metabolic consequences of oral-gut microbiome dysbiosis in type 2 diabetes, elucidating the bidirectional association mechanisms between periodontitis and diabetes. T9 Gut Microbiota and Metabolites as Mediators of Oral-Systemic Disease Connections utilizes multi-omics integrated analysis to explore the gut microbiota and its metabolites as key mediators linking periodontal disease and systemic diseases, revealing the molecular pathways mediating inflammation and metabolic disorders.

Host Immunity and Tissue Regeneration is the core objective of clinical translation, focusing on the cellular and molecular mechanisms of host immune regulation and periodontal tissue regeneration using single-cell and multi-omics technologies. T2 Stem Cell-Mediated Regeneration and Inflammation Regulation in Periodontal Disease employs single-cell RNA sequencing and transcriptomics to analyze the tissue regeneration process and inflammatory regulatory networks mediated by periodontal ligament stem cells, providing a theoretical basis for periodontal regenerative therapies. T8 Cellular and Molecular Immunology of Periodontitis: Insights from Single-Cell and Spatial Transcriptomics integrates single-cell and spatial transcriptomic technologies to map the cellular immune landscape of the periodontitis microenvironment, resolving the functional heterogeneity and molecular regulatory mechanisms of immune cell subsets, and providing new targets for precise immune interventions.

This thematic distribution reveals that research in the field of “multi-omics evolution and single-cell technologies in periodontology” is progressing from resolving the basic mechanisms of pathogen biology and virulence, to delving into the clinical translational exploration of diagnostic biomarkers in oral fluids. It expands the cross-disciplinary understanding of oral-systemic disease connections through multi-omics technologies, and ultimately utilizes single-cell technologies to resolve the precise regulatory mechanisms of host immunity and tissue regeneration. This forms a literature-derived conceptual framework from basic mechanism resolution to clinical diagnostic applications, and further to cross-disciplinary expansion and regenerative medicine translation, reflecting the field’s distinct characteristics of being guided by clinical needs, deeply integrating multi-omics and single-cell technologies, and being driven by precision diagnosis and treatment. However, this framework is based on bibliometric and topic-modeling patterns and should not be interpreted as direct experimental evidence for causal mechanisms or clinical effectiveness.

### 3.4 Analysis of key literature in the field of multi-omics evolution and single-cell technologies in periodontology

This study utilized the biblioshiny function of the Bibliometrix R package. Based on the historiographic mapping method proposed by Garfield (2004), a chronological direct citation network graph was constructed for literature related to four themes within the field of multi-omics evolution and single-cell technologies in periodontology (**Figures 5A–C**). The nodes in the historiograph represent representative papers selected based on their exploratory TSR scores. This metric was used as a supportive tool to identify potential intellectual turning points within each specific theme, rather than relying solely on global citation counts, which may be biased toward older publications. Therefore, the historiographic map should be interpreted as an exploratory visualization of citation-linked knowledge transmission within the WoSCC dataset, rather than as a definitive ranking of landmark studies.

**Figure 5:**
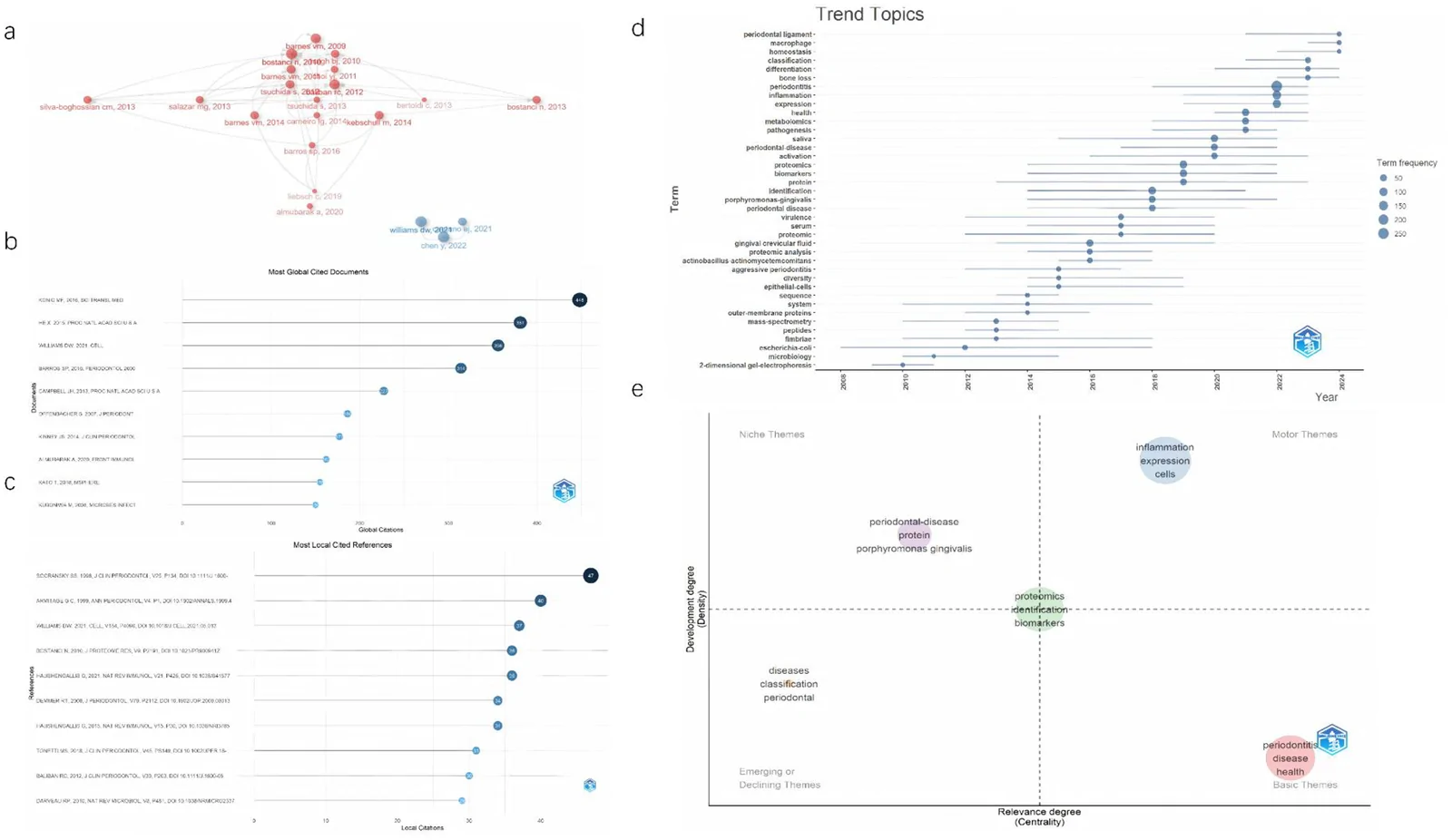
Analysis of pivotal literature on multi-omics evolution and single-cell technologies in periodontology. **(A)** Historiography results, displaying a literature analysis of representative documents selected using the exploratory Theme-Specific Ranking (TSR) approach. Each node represents information from important milestone literature. **(B)** Information and citation count for the top ten globally cited articles. **(C)** Information and citation count for the top ten locally cited references. **(D)** Evolution of research trends. Each term represents a prominent research hotspot during its corresponding time period, along with the duration of the hotspot. **(E)** Thematic Map. The graph-based community detection algorithm Walktrap reveals potentially important but relatively underdeveloped directions in the field that warrant further investigation. The X-axis indicates relevance to the field, and the Y-axis represents relative development progress.

Historiographic Evolution of the Field (**Figure 5A**). Research in the field of “multi-omics evolution and single-cell technologies in periodontology” exhibits apparent stage-evolution characteristics in the WoSCC-indexed literature. The exploration trajectories and research foci of core scholars have progressively deepened over time, forming a bibliometrically observed lineage from early exploration to mechanistic investigation, and ultimately to clinical-translation-oriented research. However, these stages reflect publication and citation patterns rather than direct experimental confirmation of a linear scientific progression.

Phase 1 (2009–2015) — Foundation of Multi-Omics Technologies and Clinical Association Exploration: Core nodes in this phase include Barnes VM (Barnes et al., 2009), Bostanci N (Bostanci et al., 2010), Tsuchida S (Tsuchida et al., 2012), Baliban RC (Baliban et al., 2012), Bostanci N (Bostanci and Belibasakis, 2012), Silva-Boghossian CM (Silva-Boghossian et al., 2013), Barnes VM (Barnes et al., 2014), and Kebschull M (Kebschull et al., 2014). Research was primarily characterized by the early application of multi-omics technologies in periodontology, focusing on the preliminary association analysis between periodontal pathogens and host molecular features. Scholars like Barnes VM pioneered transcriptomic and proteomic studies related to periodontitis, systematically resolving the molecular features of host-microbiome interactions in the periodontal microenvironment for the first time. Bostanci N’s team explored the application value of gingival crevicular fluid (GCF) proteomics in periodontal disease diagnosis, providing early evidence for non-invasive biomarker screening. Tsuchida S and Baliban RC further evaluated the potential of multi-omics technologies in elucidating the virulence regulatory mechanisms of periodontal pathogens. Driven primarily by technical methodologies, this phase established the initial application framework for multi-omics in periodontal research.

Phase 2 (2016–2020) — Deepening Mechanisms and Multi-Omics Integration: Core nodes include Barros SP (Barros et al., 2016) and Almubarak A (Almubarak et al., 2020). The research focus shifted toward the integration of multi-omics data and the in-depth resolution of periodontal disease pathology. Barros SP’s team integrated transcriptomic and metabolomic data to reveal the regulatory networks of host metabolic reprogramming and inflammatory responses in periodontitis, driving a cognitive upgrade in disease mechanisms. Furthermore, studies such as Almubarak A (Almubarak et al., 2020) highlighted the disruption of macrophage homeostasis, offering new perspectives for targeted immunomodulation. The collaborative network among scholars expanded, and the research dimension extended from single-omics characterization to multi-omics integrative analysis. These patterns suggest a literature-level shift in research emphasis, rather than direct evidence that multi-omics integration has resolved the mechanisms of periodontal disease.

Phase 3 (2021–2022) — Single-Cell Technology Breakthroughs and Clinical Translation: Core nodes such as Williams DW (Williams et al., 2021) represent cutting-edge breakthroughs and continuous exploration. Research deepened toward single-cell resolution and precision translational medicine. Williams DW and colleagues pioneered the application of scRNA-seq to study the periodontitis microenvironment, mapping the cellular heterogeneity of periodontal tissues and revealing the functional features of immune cell subsets. These achievements provided critical support for precise immune interventions and regenerative strategies, marking the field’s entry into a new era of single-cell precision analysis and clinical translation. The evolution of the collaboration network demonstrates a bibliometric pattern consistent with a paradigm shift driven by continuous cultivation and cross-team collaboration among core scholars, providing solid academic support for the precise diagnosis, targeted therapy, and tissue regeneration of periodontal disease. However, whether these research directions can be translated into validated clinical applications requires further experimental, longitudinal, and prospective clinical evidence.

Global Highly Cited Literature (**Figure 5B**). As shown in **Figure 5B**, these globally highly cited studies provide important intellectual background for the field from multiple dimensions, rather than direct bibliometric proof of clinical effectiveness: *Aggregatibacter actinomycetemcomitans*–induced hypercitrullination links periodontal infection to autoimmunity in rheumatoid arthritis (Sci Transl Med, 2016): With 448 total citations (40.73/year), this study systematically revealed the molecular association between periodontal infection and autoimmunity in rheumatoid arthritis. It provided critical multi-omics evidence for oral-systemic comorbidity mechanisms and serves as a milestone extending periodontology from localized disease to systemic health. Cultivation of a human-associated TM7 phylotype reveals a reduced genome and epibiotic parasitic lifestyle (He et al., 2015): With 381 total citations (31.75/year), this early core microbiomics study successfully cultivated human-associated TM7 bacteria. Using genomic and transcriptomic analysis, it revealed its epibiotic lifestyle, filling a critical gap in oral microbiomics and laying the methodological foundation for omics-driven research on microbial pathogenicity. Human oral mucosa cell atlas reveals a stromal-neutrophil axis regulating tissue immunity (Cell, 2021): With 356 total citations (59.33/year), this study used scRNA-seq to map the human oral mucosa cell atlas, highlighting the stromal-neutrophil axis in tissue immune regulation. It drove the study of periodontal immune mechanisms from the population level to the single-cell level. Gingival crevicular fluid as a source of biomarkers for periodontitis (Periodontol 2000, 2016): With 314 total citations (28.55/year), this study integrated proteomic and metabolomic evidence to establish the value of GCF biomarkers in disease diagnosis, monitoring, and efficacy assessment, laying a crucial foundation for multi-omics clinical translation.

Local Highly Cited Literature (**Figure 5C**). These locally highly cited documents represent important citation anchors within the present WoSCC dataset and provide a local knowledge foundation for the field. Microbial complexes in subgingival plaque (J Clin Periodontol, 1998; 47 local citations): Systematically constructed the taxonomic framework of microbial complexes in subgingival plaque, establishing the classic theoretical basis for periodontal microecological dysbiosis and providing the core framework for omics-based host-microbiome research. Development of a classification system for periodontal diseases and conditions (Ann Periodontol, 1999; 40 local citations): Standardized the clinical classification and diagnostic criteria for periodontal diseases, providing a unified evidence-based framework for clinical research and the taxonomic basis for associating multi-omics data with clinical phenotypes. Human oral mucosa cell atlas (Cell, 2021; 37 local citations): Provided crucial methodological references and data resources for local scRNA-seq-driven research on periodontal immune mechanisms. Application of label-free absolute quantitative proteomics in human gingival crevicular fluid (J Proteome Res, 2010; 36 local citations): Established a standardized proteomic system for the “gingival exudatome,” pushing periodontal precision diagnostic research from qualitative description to quantitative assessment.

Keyword trend analysis illustrates the temporal evolution of research foci, reflecting a trajectory from traditional microbiomics to multi-omics integration, and finally to single-cell precision analysis (**Figure 5D**). Early Research (2008–2013): Focused on traditional microbiology and basic proteomics. Keywords included 2-dimensional gel-electrophoresis, microbiology, fimbriae, mass-spectrometry, and outer-membrane proteins. The core was identifying virulence factors of periodontal pathogens and conducting basic proteomic analysis of GCF. Mid-term Research (2014–2018): Shifted toward omics integration and mechanistic resolution. Keywords like metabolomics, proteomics, biomarkers, gingival crevicular fluid, periodontitis, inflammation, and pathogenesis became hotspots, marking the era of multi-omics-driven molecular characterization and biomarker validation. Recent Research (2019–2024): Exhibits a trend toward single-cell breakthroughs and precision translational medicine. Emerging keywords include single-cell RNA sequencing, periodontal ligament, macrophage, homeostasis, differentiation, bone loss, and bone regeneration. This signifies a shift toward single-cell resolution, immune subset functional analysis, and regeneration-related research priorities, rather than direct confirmation of precise regenerative therapies.

Thematic Map Analysis (**Figure 5E**). Different clusters in the thematic map are distributed across a two-dimensional space based on “Relevance degree” and “Development degree,” outlining the research landscape and potential of the field. Basic Themes (High Relevance, High Development) were centered around periodontitis, disease, and health, representing the core clinical and theoretical foundation for omics applications. Basic/Technical Transition Themes (High Relevance, Medium Development) were centered around proteomics, identification, and biomarkers, suggesting continuing development in clinical validation and integrated interpretation of multi-omics data. Motor Themes (Medium Relevance, High Development) were centered around inflammation, expression, and cells, reflecting increasing attention to cellular heterogeneity and immune-subset function. Niche Themes (Low Relevance, High Development) were centered around periodontal-disease, protein, and *Porphyromonas gingivalis*, indicating a specialized focus on pathogen virulence proteins. Emerging or Declining Themes (Medium Relevance, Low Development) were centered around diseases, classification, and periodontal, suggesting that omics-assisted disease staging and classification may represent a developing area. Based on these bibliometric signals, future studies may increasingly focus on single-cell and spatial transcriptomic analyses of the periodontal microenvironment, multi-omics diagnostic models, and integrative approaches for bone resorption risk assessment. These directions should be regarded as literature-derived research priorities rather than established clinical recommendations.

**Table 2** presents the specific parameters of the top ten most highly cited articles from **Figure 5B**, including article title, journal, publication year, and total citation count.

**Table 3** presents the information of the top ten most highly cited articles from **Figure 5C**, including specific parameters such as the article title, journal, publication year, total citation count, and authors.

## 4 Discussion

Based on the above bibliometric evidence, several future directions can be framed as cautious hypotheses rather than direct conclusions. First, multicenter and large-sample multi-omics models may improve periodontal diagnosis, prognosis prediction, and treatment-response assessment compared with single-center or small-sample models, but this hypothesis requires validation across populations and regions. Second, integrating periodontal multi-omics data with oral microbiome profiles and systemic disease markers may support broader models for disease progression, treatment response, and systemic comorbidity risk; however, optimal data-integration strategies and clinically useful decision thresholds remain unresolved. Third, tailored bioinformatics algorithms and visualization tools may improve the interpretability and translational value of periodontal multi-omics data, but algorithmic performance must be tested in real-world clinical workflows. Fourth, single-cell sequencing and epigenomic technologies may help define dynamic cellular states in the periodontal microenvironment, but their translation into individualized intervention strategies remains constrained by sample acquisition, cost, data standardization, and functional validation. Therefore, these directions should be regarded as prospective research hypotheses generated from bibliometric patterns, rather than as established clinical recommendations.

The WoSCC-based bibliometric landscape of periodontology from 2000 to 2025, with early-2026 records used as an exploratory frontier update, suggests a marked shift in research emphasis from pathogen-centered profiling toward functional multi-omics and single-cell-resolution analysis of host-microbe interactions. This evolution, reflected by publication trends, keyword bursts, co-citation patterns, and LDA-derived topics, parallels the broader movement in life sciences from tissue-level averages toward higher-resolution cellular and molecular mapping. However, the present findings should be interpreted as bibliometric evidence of research activity, thematic evolution, and emerging literature trends, rather than as direct evidence of causal biological mechanisms, diagnostic performance, treatment effectiveness, or clinical utility.

Accordingly, the following discussion distinguishes four levels of interpretation: bibliometric observations derived from publication and citation patterns; literature trends reflected by keywords, topics, and co-citation clusters; biological hypotheses suggested by the broader experimental literature; and established experimental or clinical evidence reported in individual studies. This distinction is important because bibliometric analysis can reveal how a research field has evolved, but it cannot independently validate mechanistic or clinical conclusions.

### 4.1 The resolution revolution: from bulk tissue to single-cell precision

The findings from our temporal analysis suggest that Phase I and Phase II were foundational periods dominated by bulk-level transcriptomic and proteomic analyses. These methodologies provided a macroscopic molecular profile of the diseased periodontium (Demmer et al., 2008; Kebschull et al., 2014). However, bulk sequencing can obscure molecular signatures from rare or functionally specialized cell populations because signals are averaged across mixed tissues. The increased appearance of scRNA-seq-related keywords and highly cited single-cell studies in the recent period indicates that single-cell approaches have become an important methodological direction in periodontal research.

The transition to single-cell RNA sequencing (scRNA-seq) provides a higher-resolution strategy for characterizing periodontal health and disease. As highlighted in the LDA topics and keyword-burst results, landmark studies have used scRNA-seq to reconstruct the human oral mucosa cell atlas and delineate immune cell profiles associated with different periodontal states (Williams et al., 2021). These studies support a shift from purely histological or clinical-index-based descriptions toward molecular and cellular characterization. Nevertheless, the bibliometric prominence of scRNA-seq should not be equated with validated clinical utility. Bibliometric data alone cannot determine whether such cellular signatures are sufficient for clinical classification, and further experimental and clinical validation remains necessary.

### 4.2 From pathogen profiling to functional host-microbiome interactomes

Our thematic modeling indicates that pathogen biology research has developed from descriptive microbiology toward host-microbiome systems biology. Early 16S rRNA sequencing provided a foundational “parts list” of microbial communities (Socransky et al., 1998), and subsequent multi-omic profiling of uncultured organisms further expanded microbial functional understanding (Campbell et al., 2013; He et al., 2015). In the present bibliometric map, topics related to pathogen virulence, host immune response, oral-gut interactions, and metabolites suggest that the field is increasingly concerned with functional host-microbiome interactions rather than microbial composition alone.

The LDA topics related to pathogen virulence and host immune subversion (e.g., Topics 1 and 4) suggest that future studies may benefit from integrating microbial, host transcriptomic, proteomic, and metabolomic data within the same experimental framework. For example, the emergence of gut microbiota and metabolite-related topics indicates a growing interest in cross-validating metagenomic signals with systemic metabolomics. Such approaches may help clarify how localized periodontal dysbiosis is associated with broader metabolic or inflammatory changes (Hajishengallis, 2015; Kato et al., 2018). However, these interpretations should be regarded as literature-derived hypotheses and future research directions, not as conclusions directly established by the present bibliometric analysis.

### 4.3 The oral-systemic nexus: a systems biology perspective

The emergence of Domain III suggests that the oral-systemic connection has become a major research theme in the multi-omics literature on periodontology. Our bibliometric analysis identified clusters focusing on the oral-gut axis and associations between periodontitis and systemic comorbidities such as rheumatoid arthritis and diabetes. These results indicate increasing research attention to molecular links between periodontal inflammation and systemic health, but they do not by themselves establish causality.

A representative study in this domain reported *Aggregatibacter actinomycetemcomitans*-induced hypercitrullination as a molecular link between periodontal infection and rheumatoid arthritis-related autoimmunity (Konig et al., 2016). Other multi-omic studies have explored how periodontal pathogens may influence the gut microbiome and systemic metabolome (Hajishengallis and Li, 2021). These findings suggest that oral multi-omics may have potential value for systemic-risk assessment, but this possibility should be interpreted as an emerging research hypothesis supported by selected experimental studies, rather than as a conclusion directly inferred from bibliometric evidence alone.

### 4.4 Toward precision periodontology and tissue regeneration

The concentration of keywords such as “bone regeneration,” “differentiation,” and “macrophage polarization” in recent years suggests that periodontal regeneration and immune microenvironment regulation are important emerging topics. LDA Topics 2 and 8 highlight stem cell-mediated regeneration and single-cell immune mapping. Based on these bibliometric signals, future experimental studies may further examine transcriptomic transitions in periodontal ligament stem cells and immune-cell subsets under inflammatory conditions. Such work could provide candidate regulatory nodes for regenerative or immunomodulatory research, but specific therapeutic strategies require mechanistic and preclinical validation beyond bibliometric evidence.

In the diagnostic realm, studies of the “gingival exudatome” using mass spectrometry have identified candidate biomarkers in gingival crevicular fluid (Bostanci et al., 2010; Kinney et al., 2014; Barros et al., 2016). Integrating fluid biomarkers with single-cell-derived cellular signatures may provide a possible route toward molecular endotyping in periodontology. However, from a bibliometric perspective, this represents a research trend rather than an established diagnostic pathway. The translation of these biomarkers into routine precision-periodontology practice depends on external validation, standardized sampling, cost-effectiveness, and prospective clinical evaluation.

### 4.5 Future perspectives supported by bibliometric trends

The keyword-burst and thematic-map analyses suggest that single-cell RNA sequencing, microenvironmental analysis, macrophage polarization, differentiation, and bone regeneration have become active frontier signals in recent years. On this basis, future studies may prioritize three directions that are directly supported by the present bibliometric findings: first, standardized integration of multi-omics and single-cell datasets; second, functional validation of candidate immune-cell states and host-microbiome interaction pathways; and third, prospective evaluation of omics-derived biomarkers in relation to clinically meaningful outcomes such as disease activity, recurrence risk, and treatment response. However, these bibliometric signals should not be interpreted as direct evidence that specific technologies or interventions are ready for clinical implementation. Rather, they indicate areas where research attention is increasing. Spatial transcriptomics may help address the loss of anatomical context caused by tissue dissociation in scRNA-seq studies, and multimodal approaches such as scATAC-seq and CITE-seq may further connect chromatin accessibility, gene expression, and protein-level phenotypes. Nevertheless, these technologies remain technically demanding, costly, and methodologically heterogeneous in periodontal research. Future studies should prioritize standardized sampling, multimodal data integration, functional validation of candidate cell states, and links between single-cell findings and clinically meaningful phenotypes such as disease activity, recurrence risk, and treatment response. Thus, spatial and multimodal single-cell approaches represent promising research directions, but their clinical utility requires prospective validation. Artificial intelligence and machine-learning methods may support multi-omics data integration, pattern recognition, and visualization; however, their clinical value will depend on transparent algorithms, external validation, and testing in real-world periodontal datasets.

### 4.6 Synthesis of the research landscape and strategic outlook

This study highlights a bibliometric shift from bulk analysis toward single-cell-resolution research in periodontology. The integration of multi-omics and single-cell technologies has contributed to a more detailed research map of periodontitis heterogeneity, including host-microbiome interactions, immune-cell subsets, and regeneration-related pathways. These patterns provide a useful overview of how the field has evolved, but they should not be interpreted as evidence that specific technologies are already clinically mature.

Based on the integration of LDA modeling and citation-network analysis, the field appears to be moving beyond foundational molecular profiling toward more integrative and translational research questions. Future studies may prioritize cross-omics validation of biomarker panels, mechanistic investigation of cell-subset-specific pathways, and spatially informed mapping of host-microbe interactions. Only when these literature-derived directions are supported by experimental validation, multicenter cohorts, and prospective clinical studies can they contribute more directly to precision periodontology.

### 4.7 Limitations

Several limitations should be acknowledged. First, this study used WoSCC as the only data source. Although WoSCC provides standardized citation metadata suitable for co-citation, bibliographic coupling, collaboration-network, and historiographic analyses, relevant publications indexed only in PubMed/MEDLINE, Scopus, Embase, or other databases may have been missed. Therefore, the present findings should be interpreted as reflecting WoSCC-indexed periodontal research rather than the entirety of global periodontal research. Exclusive reliance on WoSCC may have influenced the observed collaboration networks, citation structures, publication trends, country and institution distributions, journal impact patterns, and the visibility of non-English or regionally published studies. Second, the language was restricted to English, which may have excluded relevant non-English studies. Third, reviews, meeting abstracts, and conference papers were excluded to focus on original research records, but this may have reduced the coverage of emerging or preliminary findings. Fourth, citation-based indicators are affected by citation time-lag, journal visibility, self-citation, and field-specific citation practices; therefore, highly cited papers should not be interpreted as automatically having greater biological or clinical validity. Fifth, bibliometric maps depend on software algorithms, parameter settings, and data-cleaning procedures, which may influence clustering, keyword co-occurrence, and network structures. Sixth, LDA topic modeling is influenced by corpus construction, stop-word selection, topic-number selection, and manual topic labeling; therefore, the nine topics identified here should be interpreted as an analytical framework rather than as fixed or exhaustive categories. Seventh, TSR was used as a study-specific composite score to support the selection of representative documents for historiographic visualization, but it is an author-developed exploratory ranking approach and has not been externally validated. Consequently, TSR should not be regarded as a standardized or universally applicable bibliometric metric, and TSR-derived historiographic interpretations should be considered supplementary and hypothesis-generating. Finally, bibliometric analysis can reveal publication patterns, collaboration structures, and emerging research signals, but it cannot establish causal biological mechanisms, diagnostic accuracy, therapeutic efficacy, or clinical utility. These questions require experimental, longitudinal, and prospective clinical validation.

## 5 Conclusion

This WoSCC-based bibliometric and scientometric analysis delineates the development of multi-omics and single-cell technologies in periodontal research from 2000 to 2025, with early-2026 records used only as an exploratory frontier update. The results suggest a transition from bulk molecular profiling and pathogen-centered analysis toward functional multi-omics, host-microbiome interaction research, single-cell-resolution mapping, and regeneration-related studies. These patterns indicate that scRNA-seq and related single-cell approaches have become increasingly prominent in the WoSCC-indexed periodontal literature, especially for identifying disease-associated cellular states and microenvironmental interactions. Meanwhile, multi-omics integration has contributed to a broader understanding of periodontal heterogeneity, oral-systemic connections, and potential biomarker discovery. However, these findings represent bibliometric trends rather than direct evidence of diagnostic validity, therapeutic efficacy, or clinical readiness. The translation of these bibliometric trends into precision diagnosis, risk stratification, targeted intervention, or regenerative therapy remains prospective and requires functional experiments, multicenter cohorts, spatial and multimodal validation, and clinically grounded outcome studies. Overall, this study provides a structured knowledge map of the field and highlights promising research directions, while emphasizing that further experimental, clinical, and multi-database validation is required before these trends can be converted into practice.

## Data Availability

Publicly available datasets were analyzed in this study. The datasets analyzed for this study were retrieved from the Web of Science Core Collection (WoSCC). The raw bibliometric data (plain text files) supporting the conclusions of this article will be made available by the authors, without undue reservation, upon reasonable request to the corresponding authors (Lirong2026@dali.edu.cn).
